# Editor's notes

**DOI:** 10.3402/jchimp.v3i1.20824

**Published:** 2013-04-17

**Authors:** Robert P. Ferguson

In this issue of the journal, we have 10 manuscripts. There are two very interesting opinion pieces in editorial form. In our previous issue, a survey by Buscemi resulted in questions regarding the necessity of the maintenance of internal medicine certification by the American Board of Internal Medicine's (ABIM) Maintenance of Certification Program. Dr. Kempen expands on this and is in strong support of reducing requirements of maintenance of certification status. A second editorial comments on an article in the same issue, on the variable of patient lifestyle (rather than medical care) influencing medical readmissions. Dr. Gulati comments on the implications financially. There are five case reports, plus an imaging study. Two case reports have iatrogenic origin, a case of atrioventricular ablation complicated by phrenic nerve palsy, and an esophageal obstruction complicating ligation of esophageal varices. There is also a case of pulmonary emboli causing false-positive elevations in cardiac enzymes, as well as a case of delayed response of recurrent syncope secondary to vagal tone after three decades. There is another case demonstrating the effect of diabetes on muscle anatomy. There are two research studies: one points out that CT scans for pulmonary emboli may be overdone and inaccurate and that we could do better by reducing our dependence on them. A second research study looks at computer software as part of a weight control program. There is also an imaging paper, showing diagnostic criteria for empyema of the lung and the importance of early intervention when patients meet the diagnostic criteria.

*Robert P. Ferguson, MD* Editor
